# Towards Interactive Search: Investigating Visual Search in a Novel Real-World Paradigm

**DOI:** 10.3390/brainsci10120927

**Published:** 2020-12-01

**Authors:** Marian Sauter, Maximilian Stefani, Wolfgang Mack

**Affiliations:** 1 General Psychology, Bundeswehr University Munich, 85579 Neubiberg, Germany; maximilian.stefani@unibw.de (M.S.); wolfgang.mack@unibw.de (W.M.); 2 General Psychology, Ulm University, Albert-Einstein-Allee 47, 89081 Ulm, Germany

**Keywords:** interactive search, visual search, real-world, action intentions, haptic

## Abstract

An overwhelming majority of studies on visual search and selective attention were conducted using computer screens. There are arguably shortcomings in transferring knowledge from computer-based studies to real-world search behavior as findings are based on viewing static pictures on computer screens. This does not go well with the dynamic and interactive nature of vision in the real world. It is crucial to take visual search research to the real world in order to study everyday visual search processes. The aim of the present study was to develop an interactive search paradigm that can serve as a “bridge” between classical computerized search and everyday interactive search. We based our search paradigm on simple LEGO^®^ bricks arranged on tabletop trays to ensure comparability with classical computerized visual search studies while providing room for easily increasing the complexity of the search environment. We found that targets were grasped slower when there were more distractors (Experiment 1) and there were sizable differences between various search conditions (Experiment 2), largely in line with classical visual search research and revealing similarities to research in natural scenes. Therefore, our paradigm can be seen as a valuable asset complementing visual search research in an environment between computerized search and everyday search.

## 1. Introduction

We are all experienced searchers. We have searched for our keys, an important note on the desk or even a familiar person in a crowd. Searching is so central that it is even assumed that visual search is the most prevalent task our visual system has to engage in [1]. We have to search because we simply lack the capacity to identify our entire environment at once [1]. While early visual search experiments were presented on a tachistoscope [2], an overwhelming majority of visual search studies nowadays have been conducted using computer screens [3]. These all make use of peripheral response mechanisms that do not involve direct interaction with the search targets. In these computerized experiments, research often relies on simple static stimuli in extremely simplified search paradigms [2] that hardly resemble everyday search situations. In day-to-day life, however, we search at a different time scale, that is, seconds or even minutes, rather than milliseconds, search targets are less well defined [4] and search takes place in far more complex spatial arrangements and is interactive (i.e., involves grasping, head or body movements). As such, there are certainly shortcomings in transferring knowledge from computer-based visual search studies to real-world search behavior. The need to employ more naturalistic paradigms has recently been recognized in experimental psychology in general [5,6,7,8,9]. The fundamental discrepancy between classical laboratory research and real-world search behavior has been identified and can be addressed by two general approaches: (1) investigating real-world behavior and simplifying paradigms afterwards (cognitive ethology, [7]) and (2) extending classical visual search research by, for example, the employment of naturalistic scenes [10].

At the core of cognitive ethology is the philosophy that in order to study real-world behavior, researchers ought to first observe the behavior of interest in everyday, real-world settings and only subsequently impose small restrictions on the tasks or environment in order to isolate cognitive processes that are common across situations. Since its suggestion in 2008, there have only been a few visual search investigations in a largely uncontrolled environment [11,12,13,14,15,16]. For example, Riggs and colleagues [14] investigated how people search in a natural open terrain task. Participants had to search for coins in a grassland environment and report which side of the coin was facing up. They found that the search strategy is an important determinant of success in real-world search, which is typically not observed in simple search tasks, possibly because in such tasks, the search only takes some hundreds of milliseconds and search targets are salient. In another study [15] in which participant dyads searched for hidden objects in an indoor scene, the authors found that locations that were investigated once were almost never revisited (a finding confirming earlier real-world research [17]), quite unlike in artificial searches [12].

In classical computerized studies using simple search displays, targets and distractors can typically be differentiated without much effort by comparison in only one visual dimension, like color, shape or orientation (feature searches). For example, the search target is a diamond while all the distractors are squares. This type of search is considered to be fast because the target is very prominent and pops out among the distractors [2,18]. All items in the search scene can be scanned in parallel. This implies that response times to these types of visual scenes do not increase when the set size increases (i.e., more items are in the search display). In a slightly more complex search, the target differs from the distractors by a conjunction of two or more basic features (feature conjunction search). For example, the distractors are either large squares or small triangles, while the search target is the only large triangle. In this case, the target does not pop out among the distractors and attention is required to guide the search serially through the display to find the conjunction of features that define the target. This crucial manipulation of the set size and its differential effect on the search slope between (most) features and feature conjunction searches are some of the most prominent and replicated effects in the visual search literature [1,2,18], but they can only be replicated in a real-world setting if objects are easily separable and there is reliable control over the scene’s set size.

In an effort to bring classical visual search research closer to the real world, researchers have recently employed natural scenes in computerized searches, presented as static pictures [10,19,20,21]. For example, Neider and colleagues [21] asked participants to look for tanks and helicopters in quasi-natural scenes of outdoor environments (sky above grasslands with trees) and found that search efficiency increased when more trees were added. This is a surprising finding considering search efficiency typically quickly decreases in classical visual searches (or remains unaffected in very simple pop-out searches) [2,18]. The authors interpret their results by discussing the traditional conceptualization of “set sizes” (i.e., the number of objects relevant to the search) and their applicability to naturalistic scenes. How do we know what an object is? Is a tree a single object? Or is each separate branch an object? Or each leaf? This uncertainty means that we cannot reliably estimate the number of separate items in the scene [22,23]. This is a problem as search efficiency is typically (but not always [24]) investigated by looking at the search slope (search time per item) when the set size increases [18].

Another criticism of visual search research in naturalistic scenes is that it is based on static picture viewing [25]. This does not compare well with the dynamic and (inter)active nature of vision in the real world [5]. The interaction between (visual) attention and physical actions (e.g., hand movements) makes up one of the most important facets of our everyday life, but cannot be investigated in an ecologically valid manner by utilizing predominantly static images. However, this is crucial, as actions or even mere action intentions can modulate perception, the so-called action–perception transfer (APT; [26]), which has since been confirmed in various domains (for a review, see [27]). One study found that eye movements differ in an only-looking task compared to a looking-and-tapping task [28], indicating that interacting with objects might change basic visual search processes. In another study, participants had to either point and look at a target or grasp the target [29]. The authors found that participants executed fewer saccades to wrongly oriented (i.e., distracting) objects in the grasping condition compared to the pointing condition, while saccadic latencies remained similar. This suggests that specific action intentions (i.e., grasping) can enhance visual processing, at least for action-relevant features, like orientation. However, Solman and colleagues [30,31] conducted interactive (computerized) search studies, in which participants had to manipulate and visually identify targets that were arranged on a virtual “pile”. They found that some targets were manipulated but not identified and had to be revisited. They concluded that perception not necessarily directs action but rather acts as a more general supervisory system. In particular, they postulate that processes involved in “perception for action” and “perception for identification” may operate independently even when tight coordination would be beneficial.

The aim of the present study is to explore how we could potentially bridge the gap between classical visual search research and novel real-world search by introducing a flexible, adaptive paradigm that offers enough standardization for comparisons with classical research while providing flexibility in terms of how complex the search task and environment can be realized. Therefore, we conducted an interactive search, in which the search targets not only have to be found but also moved (i.e., grasping movements have to be prepared), situated in the real world using simple objects to maximize the comparability with classical computerized visual search studies. In order to maximize the number of trials while accounting for the pragmatics of needing to manually prepare the search spaces, a foraging-type design in which targets are present in all search spaces and multiple targets need to be found in the same search space was deemed most appropriate (c.f. [32,33] for similar computerized designs). We specifically focused on how the variation in set sizes influences response times in feature conjunction searches in an interactive real-world setting (Experiment 1) and how this (optimized) setting can reveal systematic differences between feature and feature conjunction searches (Experiment 2). The overarching goal was to provide a bridging paradigm that can be approached from both sides of the “lab-to-everyday-search” scale: classical visual search researchers trying to generalize findings to the real world as well as cognitive ethologists wishing to impose restrictions on everyday search.

## 2. Experiment 1

In the first experiment, we transferred a classical feature conjunction search into the real world using a variety of LEGO^®^ bricks arranged on trays. Our initial approach was to use bags of pieces like they arrived in our lab, to derive the first result without too much incidental effort in creating the search spaces—allowing for an easy adoption. With this setup, we aimed to show the set size effect that is typically revealed in feature conjunction searches: When more distractors are present in the visual scene, participants need longer time to search for the target items, while the search time scales linearly with the number of distractor items. We would like to emphasize that this investigation is to be taken as explorative with regard to the outcome and reliable estimates of statistical power cannot be generated due to the novel nature of the paradigm. The analyses therefore stress effect sizes and confidence intervals rather than *p*-values.

### 2.1. Methods

#### 2.1.1. Participants

A total of 20 participants were tested (7 female, 13 male; 19–30 years old, M = 23.2, SD = 3.2). All of them had normal or corrected-to-normal vision. Accurate color vision was ensured with a 10-plate Ishihara (1979) color test. They were naïve to the purpose of the study and provided written informed consent. All participants were students from the Bundeswehr University Munich. The study was conducted in accordance with the Declaration of Helsinki and the ethics committee of the Bundeswehr University Munich.

We assessed the experience participants had with handling the LEGO^®^ bricks. On a five point scale with the choices from (1) “I have no experience with handling such bricks” to (5) “I have a lot of experience with handling such bricks” (translated from the original German), the participants came to an average of 3.15 (SD = 1.27). Most participants had an average experience handling such bricks and the variance was quite small.

#### 2.1.2. Materials

For this experiment, we used pre-packaged sets as they arrived at our laboratory. In particular, we took the pre-packaged sets from ten LEGO^®^ 10698 Classic boxes. The pieces are available in 33 colors and grouped by color similarity in small plastic bags (e.g., yellow and orange pieces constituted one bag). On average, there are 101 different pieces (SD = 10.53) in one bag, deviating in color, shape and size. Six different color families (“color sets”) were used: yellow/orange; light/dark blue; pink/purple; red/dark brown; beige/light brown; and white/grey.

**Figure 1:**
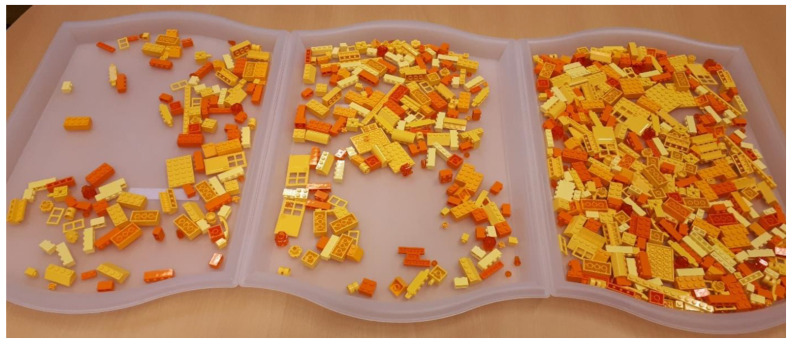
The three yellow/orange search trays. From left to right: easy (~100 pieces), medium (~200 pieces) and difficult (~400 pieces). Note that they were not presented next to each other to the participant but on top of each other in order to limit visibility of the next search tray.

Three search trays were prepared for each color set. The first search tray consisted of one pre-packaged bag (about 100 pieces, easy search difficulty), the second consisted of two bags (about 200 pieces, medium search difficulty) and the third consisted of four bags (about 400 pieces, hard search difficulty). To enable a quick switch between search tasks, all search tasks were prepared on colorless trays measuring 52 × 35 cm (Figure 1). The pieces were not mounted to specific positions within the search trays and the arrangement was randomized between participants by shuffling all pieces by hand.

#### 2.1.3. Procedure

The participants’ task was to find nine target objects (e.g. all nine yellow 4-cubes) within a search tray as quickly as possible without wrongly placing a distractor outside of the tray. Two runs were carried out. For the two runs, there were two different target objects for each of the search trays (e.g., for the yellow/orange set, the first-run targets were 2 × 2 yellow bricks and second-run targets were 1 × 4 orange bricks). They were prepared on small green plates that were placed to the left of the tray and shown to the participants before starting a new color set. The order of the color sets, as well as the difficulty conditions, was determined randomly and differently for each participant. The response time assessment was programmed with OpenSesame 3.2.2 [34].

The search began with the start signal of the experimenter by clicking the left mouse button. The participants had to find all the targets as quickly as possible and remove them from the tray using their dominant hand. With their non-dominant hand, a USB pointer was operated to measure the time to find each target. Participants were instructed to click the pointer at the exact time when they placed a piece next to the tray (as opposed to on pickup). We chose clicking-at-placing over clicking-at-pickup because earlier testing had indicated that participants can coordinate clicking better by placing the pieces (possibly because clicking felt more natural when the task was “completed”). While this approach leads to a slight overall increase in response times, errors could be completely eliminated (as wrongly touching a distractor is not punished).

Importantly, the participants had to search for and grasp the nine targets one after the other. They were not allowed to hold more than one target in their hand at a time. The participants were free to choose where they wanted to place the target pieces (in front of or next to the tray), to minimize the cognitive resources spent to choose a place. After the participant had found all nine targets, the trial ended and all nine targets were mixed into the distractors again. The trays within one color category were always arranged from easy to hard and were placed on top of each other, so that only the currently searched tray was visible for the participant. All other trays were kept outside the participant’s visual field behind a cover, so that prior peeking of future search trays was not possible. The order of the color sets varied randomly between participants. After all search trays were completed for the first time, they were prepared again for the second round (outside of the visual field of the participant), in which participants had to search for new target objects, but the rest stayed the same. In total, a participant searched 324 times in this experiment.

### 2.2. Results

**Figure 2:**
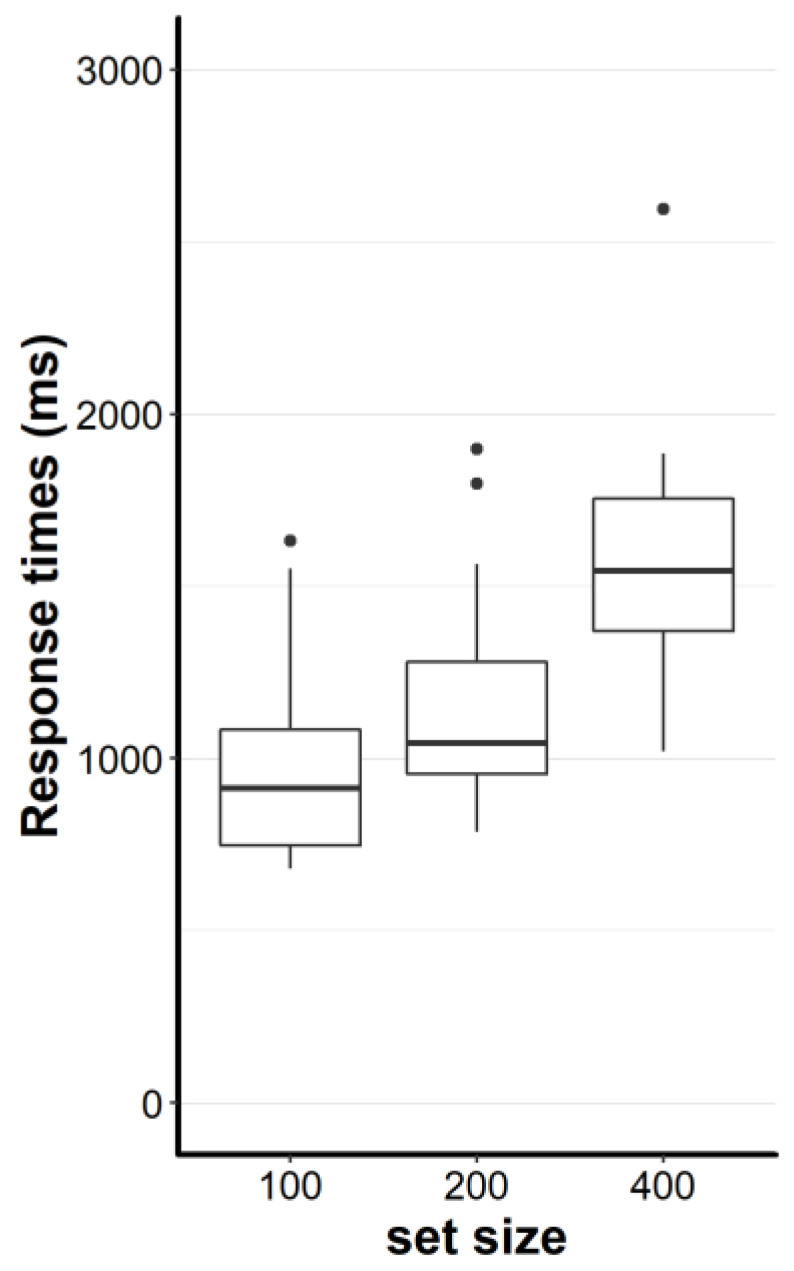
Median response times for the easy condition (set size 100), medium condition (set size 200) and hard condition (set size 400). The lower and upper hinges (boxes) correspond to the first and third quartiles (the 25th and 75th percentiles). The upper whisker extends from the hinge to the largest value no further than 1.5 ×IQR from the hinge (where IQR is the inter-quartile range, or distance between the first and third quartiles). The lower whisker extends from the hinge to the smallest value at most 1.5 × IQR of the hinge. Data beyond the end of the whiskers are called “outlying” points and are plotted individually (black dots).

We calculated median response times and submitted them to a repeated measures ANVOA with the factor set size. As expected, the set size main effect was large: F, (2, 38) = 28.7, *p* < 0.001, η_p_² = 0.6 (see Figure 2). Subsequent paired t-tests showed that the average response time was shorter in the easy condition compared to the medium condition, M = −197 ms; t, (19) = −8.67, *p* < 0.001, d_z_ = 1.94, 95% CI [−245 ms, −149 ms]. The response time was on average shorter in the easy condition compared to the hard condition, M = −752 ms; t, (19) = −5.97, *p* < 0.001, d_z_ = 1.34, 95% CI [−1015 ms, −488 ms]. The t-test between the medium and the hard condition also shows a sizable difference, M = −554 ms; t, (19) = −4.47, *p* < 0.001, d_z_ = 1.0, 95% CI [−814 ms, −295 ms]. Taken together, all estimates of effect sizes are to be considered large (all d ≥ 1.0) and none of the confidence intervals include zero.

### 2.3. Discussion

The purpose of the first experiment was to reveal the set size effects typically found in visual conjunction searches by using an interactive paradigm based in an interactive real-world setting. We could show that, across all color sets, there are large set size effects. There was an increase in response times per item which scaled linearly with the numbers of distractors in the visual scene. Using this setup, we could show that in complex searches, each distractor contributes to the overall search time reducing efficiency in the search. This is generally in line with classical research showing set size effects in on-screen visual search (c.f. [1,35]).

Expectedly, response times per target item are higher than in comparable laboratory studies due to the additional physical effort but the variance in the data is quite low, so there is likely not much variance introduced from grasping motions. The effect is consistent across all participants.

A caveat of this approach was that the participants timed themselves using a pointer, which led to confusion in some participants as they forgot to click the pointer or accidentally clicked too many times. To develop a more consistent setup, it is therefore necessary to not let the participant time themselves, but rather have the experimenter or a computer-based system control the timing.

## 3. Experiment 2

In Experiment 2, we assessed differences between feature searches and conjunction searches. We chose to investigate two feature dimensions, namely color and size, as well as their feature conjunction because these dimensions are often used in classical laboratory studies. While there seems to be consistent evidence that clear color differences between the search target (e.g., red) and the rest of the objects (e.g., blue) will make the target “pop out” of the search scene, leading to easy searches [18], it is less clear how large size differences have to be in order to make individual pieces pop out. In an initial piloting with two participants, we found that searching for “2-knob” differences, e.g., pieces with 4 knobs (squares) compared to pieces with 6 knobs (rectangle), was comparably slow in an unordered setting. This is why we thought that color searches would be the easiest type of search, shape searches would be the hardest and conjunction searches would fall in between. Notably, in this setting, a shape difference always goes along with a size difference and cannot be distinguished since there are no “bigger” LEGO^®^ bricks, which are still similar in all other dimensions. Exploratively, we further investigated potential differences between searches guided by a grid compared to unguided searches. The motivation for this was that, previously, it was found that grid-like structured search spaces may alter (and possibly improve) performance in the search [36] and a search strategy in general can be beneficial for search performance [14,15]. As in Experiment 1, the analyses stress effect sizes and confidence intervals rather than *p*-values due to the explorative nature of this investigation.

### 3.1. Methods

#### 3.1.1. Participants

A total of 37 students participated in this experiment (9 female), aged between 20 and 33 (M = 24, SD = 3.5). All of them had normal or corrected-to-normal vision. Accurate color vision was ensured with a 10-plate Ishihara (1979) color test. They were naïve to the purpose of the study and provided written informed consent. All participants were students from the Bundeswehr University Munich. Again, we assessed the experience participants had with handling the bricks. On a five-point scale with the choices from (1) “I have no experience with handling such bricks” to (5) “I have a lot of experience with handling such bricks”, the participants came to an average of 2.85 (SD = 1.10). Thus, most participants had an average experience handling the bricks and the variance was quite small. The study was conducted in accordance with the Declaration of Helsinki and the ethics committee of the Bundeswehr University Munich.

#### 3.1.2. Stimuli

**Figure 3:**
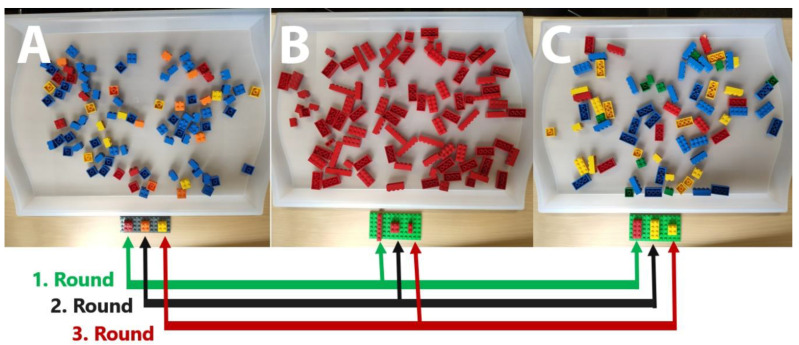
Examples of the search trays for color searches (**A**), shape searches (**B**) and conjunction searches (**C**). Participants completed three rounds of 20 trays, while in the first round, they searched for all leftmost targets (green arrows), then all middle targets (black arrows) and then all rightmost targets (red arrows). The order was counterbalanced.

For the study, 20 different search trays were developed, resembling searches in which the search target could be singled out by shape/size or color (5 each) or their conjunction (10). They were presented in a random arrangement with the exception that feature and conjunction searches were always alternating. Each search tray consisted of the same components: a flat, transparent tray measuring 3 × 52 × 35 cm, 30 target objects (3 target types) and a number of 40 to 60 distractors adapted to the type of search (c.f. Figure 3). Note that by using LEGO^®^ bricks, it is not possible to change the shape of a brick without also changing its size and vice versa. The differential instances of objects in the shape/size feature condition always represent changes in both dimensions, but will further be called “shape” for simplicity.

#### 3.1.3. Setup and Procedure

To further illustrate the procedure, we uploaded an example video of one of the participants (see Open Practices statement). The search trays were presented on three rows of tables, with seven trays in the first row, six in the second row and seven in the third row. Each search tray was aided by an external plate on which the three targets were plugged so that the participant could see and compare their current target at any time. On the wall in the direction of the test person′s gaze, a photo and the constantly updating number of already found targets per run were displayed with a projector. When a respondent found the ten targets on a tray, a beep sound occurred. The participant always stood at the side of the table where the external plate was located and held a plastic box in their weak hand. This was used to collect the found search targets. The experimenter stood directly opposite them and instructed which target to search for (the participant had to point at it to avoid left–right confusions) and that all targets had to be picked up and placed in the box one by one. For each search, the experimenter only revealed the tray the participant was using. All other trays were hidden under a gray plate. The experimenter gave the signal to begin the search by saying “start” and uncovering the search tray in front of the participant. Response times were measured by the experimenter through clicking a button on a pointing device for each search target. This means the first response time was measured from the “start” signal until the first target item was placed in the box. When the participant had removed the ten targets from the tray, a stop signal sounded. The participant then put the targets back into the tray. The experimenter shuffled all the pieces, covered the tray again and continued with the participant to the next search tray. The participant was instructed to work the trays from the left tray to the right tray in each row and from the front row to the back row. During the first pass, the participant searched for the leftmost targets (or rightmost; counterbalanced) shown on the external plate on all trays. On the second pass, they searched for the middle targets and on the third pass, the remaining left or right target. The search targets and distractors were mixed unsystematically on the trays. In the grid search condition, the trays were prepared with white adhesive tape that divided the search tray in 6 roughly equally sized zones (see Supplementary Materials).

### 3.2. Results

**Figure 4:**
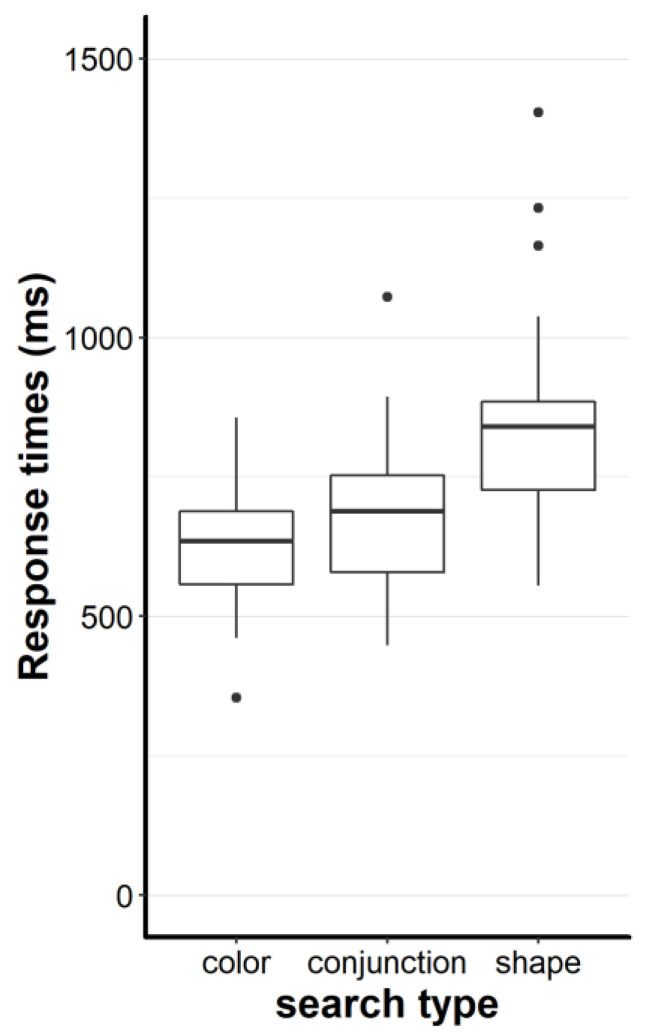
Response times as a function of the search type. The lower and upper hinges (boxes) correspond to the first and third quartiles (the 25th and 75th percentiles). The upper whisker extends from the hinge to the largest value no further than 1.5 × IQR from the hinge (where IQR is the inter-quartile range, or distance between the first and third quartiles). The lower whisker extends from the hinge to the smallest value at most 1.5 × IQR of the hinge. Data beyond the end of the whiskers are called “outlying” points and are plotted individually (black dots).

In Experiment 2, we assessed systematic differences between feature and feature conjunction searches in an interactive real-world setting. To investigate this, we first contrasted response times in feature searches vs. feature conjunction searches. As we did not find any differences between grid searches and non-grid searches (p > 0.1), we further report the averages of all searches. We submitted the median response times (RTs) to a repeated measures ANOVA with the factor search type (color, shape, conjunction). The search type main effect was quite large: F, (2, 72) = 121.97, *p* < 0.001, η_p_^2^ = 0.77 (c.f. Figure 4).

Subsequent Welch’s t-tests showed that conjunction searches tended to be slower than color searches, 678 vs. 628 ms: t, (70.12) = 1.8, *p* = 0.076, d_z_ = 0.42, 95% CI [−5 ms, 105 ms], conjunction searches were generally faster than shape searches, 678 vs. 842 ms: t, (64.78) = −4.5, *p* < 0.001, d_z_ = 1.05, 95% CI [−237 ms, −91 ms], and color searches were faster than shape searches, 628 vs. 842 ms: t, (58.88) = −6.16, *p* < 0.001, d_z_ = 1.43, 95% CI [−283 ms, −144 ms]. Effect sizes range from medium to large and one confidence interval includes zero. This indicates that differentiating feature searches from feature conjunction searches needs careful consideration in terms of the chosen feature dimensions and their discriminability.

**Figure 5:**
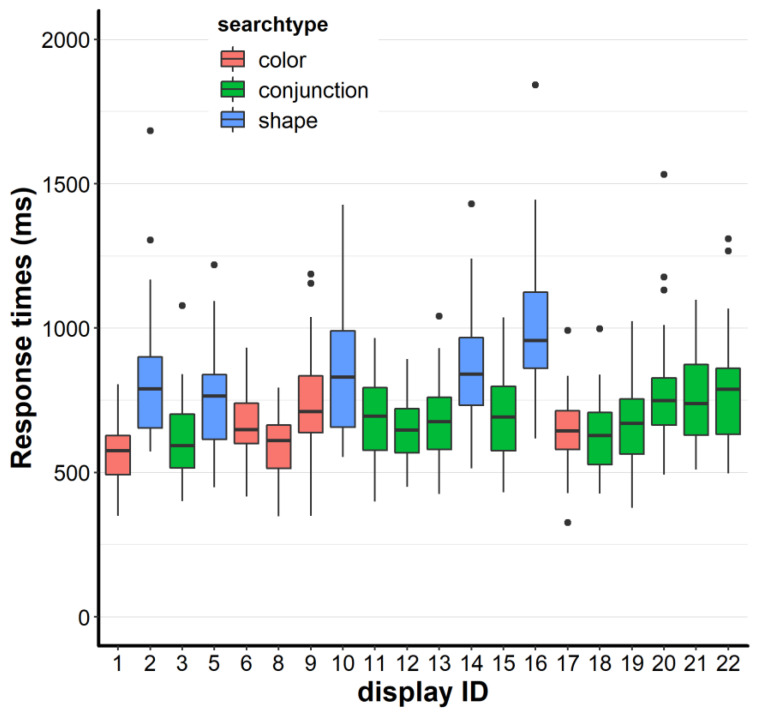
Response times as a function of the tray IDs. The lower and upper hinges (boxes) correspond to the first and third quartiles (the 25th and 75th percentiles). The upper whisker extends from the hinge to the largest value no further than 1.5 × IQR from the hinge (where IQR is the inter-quartile range, or distance between the first and third quartiles). The lower whisker extends from the hinge to the smallest value at most 1.5 × IQR of the hinge. Data beyond the end of the whiskers are called “outlying” points and are plotted individually (black dots).

Looking at the individual trays (Figure 5), one can see that shape searches led to (visually) higher variance. In particular, tray 16 (for a visual, see Supplementary Materials) seemed to be an unordinary difficult search. In fact, the averaged other shape searches were faster compared to tray 16, 814 vs. 994 ms: t, (68.31) = −3.68, *p* < 0.001, d_z_ = 0.86, 95% CI [−276 ms, −82 ms]). However, upon visual inspection, it seems that search type was the prime determinant of search time as the shape searches were all comparatively rather slow and the color searches were all rather fast. Notably, if tray 16 was left out of the search type comparison, conjunction searches were still faster than shape searches, 678 vs. 814 ms: t, (64.32) = −3.71, *p* < 0.001, d_z_ = 0.86, 95% CI [−210 ms, −63 ms], but the effect size decreased (compared to the original d_z_ = 1.05).

### 3.3. Discussion

In the second experiment, we investigated the difference between color, shape and conjunction searches in an interactive real-world setting. In our paradigm, we found that feature searches were fast (color) or slow (shape) depending on the discriminability of the items within each feature dimension, i.e., the differences in stimulus color were quite easily noticeable, while the shape/size changes were harder to identify. The feature conjunction searches were in the middle in terms of response times. This is at odds with the computerized search literature, in which it is typically found that conjunction searches are generally slower than feature searches [2,18]. An explanation could be that participants were able select the respective stimulus color in a parallel fashion and the subsequent inefficient shape search was limited to a smaller subset of the original search space. Interestingly, from visual inspection, it seems that there is not too much variance in response times to the individual trays for color searches as well as feature conjunction searches (except for a few outliers).

## 4. General Discussion

In these experiments, we developed an easy-to-adopt yet standardizable paradigm to investigate common visual search questions incorporating haptic components vital for comparison to applied search tasks in the real world. In the first experiment, we investigated the search efficiency of conjunction searches and were able to show classical linear set size effects: average response times to targets were higher as a function of set size, which is in line with prior laboratory research [1,18,35]. This shows that it is, in principle, possible to generalize set size effects found in classical computerized experiments to the real interactive world.

In the second experiment, we showed that feature searches in the color dimension were faster than conjunction searches. This is theoretically in line with prior computerized studies in the sense that color differences (if large enough) result in a strong bottom-up salience signal [2,18] and thereby are a quite prominent attribute guiding the search [37]. However, shape searches were slower than conjunction searches in the present study. This suggests that, in our paradigm, shape was not a strong attentional guiding attribute. In fact, shape seemed to be so poor at guiding the search that, in conjunction searches, participants seemingly selected all colored target objects first and then performed the apparently much harder shape search on a smaller subset of items, leading to a net RT benefit compared to ordinary shape searches. A reason for this might be that the shape differences were perceived as smaller than the differences in color (c.f. Figure 3), which might explain why this trend can generally be found for all shape search trays (visual inspection of Figure 5). Notably, even in computerized experiments, shape searches tend to be slightly slower than color searches, especially with a set size of 30+ items [38]. It is reasonable to assume that shape differences would lead to similarly easy searches, if the shape differences are only large enough to make the search targets pop out of the search space, for example, if spheres were contrasted with squares. Relatedly, participants might have been able to categorize and verbalize the color differences (e.g., search for blue among yellow and orange) but not the shape differences (shape targets were all somewhat cube-ish). Classical computerized search studies typically relied on clearly distinguishable categories for shape searches as well (e.g., search for a diamond among squares), and while there is evidence that shape information can be beneficial for guiding attention in the search [39], this is not undoubted [37] and it could be that shape is only a strong guiding attribute when verbal categories can be established. However, a computerized study investigating attentional dynamics in a color–shape conjunction task found that while color distractors reliably captured attention, while shape distractors did not, although they were defined in a verbalizable category [40]. Overall, it seems that color is an arguably stronger guiding attribute than shape and our study might have pronounced that difference.

From a data perspective, our response time results can best be compared to the computerized finger-based foraging tasks popularized by Kristjansson et al. [32,33]. In these tasks, participants searched for feature or conjunction targets by cancelling the targets through pointing at them on a tablet device. In our study, response times in color feature searches were ~628 ms per target (compared to ~298 ms [33] and ~315 ms [32]) and response times in conjunction searches were ~678 ms (compared to ~350 ms [33] and ~440 ms [32]). This means, in the computerized foraging tasks, conjunction responses were 1.4 times slower than feature searches (and this is quite typical for computerized search tasks [1,2,18]), while there were no differences found in the present study. This is likely because responses in our shape search condition were particularly slow. It is plausible that the feature conjunction searches (color × shape) were found at medium response times (between fast color and slow shape searches) because participants first singled out all correct colors in parallel, reducing the functional set size [21] of the search space [2,18] (benefitting from the color pop-out effect), and then had to effortfully search through the differently shaped objects (but limiting the inefficient serial search to a subset of the original search space).

A difference to computerized studies remains in the fact that in this interactive unstructured setting, there is more than one “target”—strictly speaking—because the target can be present in almost every imaginable orientation. These orientation changes include the fact that sometimes the “back” of the target might face the observer, so the target cannot be directly identified by its number of knobs. This might be a reason why color is the prominent driver of search times: it is static across all possible target orientations. However, the shape or size of the target, usually identified by the number of knobs, has to be identified by the number of “gaps” instead when the target is laying on its “belly”. It can be argued that a new mental representation of the target is needed to successfully find the “gaps” corresponding to the correct shape of the target, which would mean that for shape searches, participants would have to hold multiple target representations in their visual working memory. There has been a discussion in the search literature whether an observer can hold multiple target templates in their visual working memory at the same time (yes: [41]; no: [42]; divergent: [43]) or whether templates need to be costly “switched”. Therefore, it might be that shape searches are not only slower because of decreased discriminability between target and distractors, but also because multiple target representations have to be effortfully held in visual working memory. Further research might compare similar unstructured settings with more orderly presented targets in this real-world paradigm to further inform this issue.

Currently, our novel paradigm probably resembles lab-based research more closely than true everyday real-world search behavior. However, we argue that it is provides valuable benefits and complements classical research. Using this paradigm might further address the issue of ambiguous set sizes in real-world settings [22,23]. It is therefore straightforward to calculate search efficiency in controlled stimulus arrangements. This makes search in the real world comparable to computerized search studies (c.f. 19). Additionally, we introduced an interactive, haptic component to the search that is prevalent in nearly all everyday searches and therefore vital towards a more complete understanding of human search behavior in applied settings.

Overall, the present article provides two studies that aid towards an ecologically valid demonstration of crucial effects in line with the feature integration theory that much of todays’ visual search literature relies upon [2]. It describes a novel paradigm to investigate visual search effects in an interactive real-world setting and might serve as a “bridge” between classical computerized search and everyday interactive search.

## Supplementary Materials

The data and analysis scripts for both experiments are available at https://dx.doi.org/10.17605/OSF.IO/AQ4NM. The example video of the experimental procedure is also available at the above link. Note that to protect the privacy of the persons in the video, we have limited the video to the first round, cut the video slightly and removed the audio. None of the experiments were preregistered.
